# Evaluation of rRNA depletion methods for capturing the RNA virome from environmental surfaces

**DOI:** 10.1186/s13104-023-06417-9

**Published:** 2023-07-07

**Authors:** Yuh Shiwa, Tomoya Baba, Maria A. Sierra, JangKeun Kim, Christopher E. Mason, Haruo Suzuki

**Affiliations:** 1 Department of Molecular Microbiology, Tokyo University of Agriculture, Tokyo, Japan; 2 NODAI Genome Research Center, Tokyo University of Agriculture, Tokyo, Japan; 3 Advanced Genomics Center, National Institute of Genetics, Mishima, Japan; 4 Joint Support-Center for Data Science Research, Research Organization of Information and Systems, Tokyo, Japan; 5 Tri-Institutional Computational Biology & Medicine Program, Weill Cornell Medicine, New York, NY USA; 6 Department of Physiology and Biophysics, Weill Cornell Medicine, New York, NY USA; 7 The HRH Prince Alwaleed Bin Talal Bin Abdulaziz Alsaud Institute for Computational Biomedicine, Weill Cornell Medicine, New York, NY USA; 8 The WorldQuant Initiative for Quantitative Prediction, Weill Cornell Medicine, New York, NY USA; 9 Institute for Advanced Biosciences, Keio University, Tsuruoka, Yamagata Japan; 10 Faculty of Environment and Information Studies, Keio University, Fujisawa, Japan

**Keywords:** rRNA depletion, Virus detection, Environmental metatranscriptome analysis, Virome, Library preparation

## Abstract

**Objective:**

Metatranscriptomic analysis of RNA viromes on built-environment surfaces is hampered by low RNA yields and high abundance of rRNA. Therefore, we evaluated the quality of libraries, efficiency of rRNA depletion, and viral detection sensitivity using a mock community and a melamine-coated table surface RNA with levels below those required (< 5 ng) with a library preparation kit (NEBNext Ultra II Directional RNA Library Prep Kit).

**Results:**

Good-quality RNA libraries were obtained from 0.1 ng of mock community and table surface RNA by changing the adapter concentration and number of PCR cycles. Differences in the target species of the rRNA depletion method affected the community composition and sensitivity of virus detection. The percentage of viral occupancy in two replicates was 0.259 and 0.290% in both human and bacterial rRNA-depleted samples, a 3.4 and 3.8-fold increase compared with that for only bacterial rRNA-depleted samples. Comparison of SARS-CoV-2 spiked-in human rRNA and bacterial rRNA-depleted samples suggested that more SARS-CoV-2 reads were detected in bacterial rRNA-depleted samples. We demonstrated that metatranscriptome analysis of RNA viromes is possible from RNA isolated from an indoor surface (representing a built-environment surface) using a standard library preparation kit.

**Supplementary Information:**

The online version contains supplementary material available at 10.1186/s13104-023-06417-9.

## Introduction

A growing interest in the presence of microbes and viruses on built-environment surfaces [1] has encouraged the international consortium MetaSUB [2] (established in 2015) to investigate the urban microbiome on a global scale. Approximately 5,000 samples obtained from built-environment surfaces (such as subway stations) in 60 cities worldwide were subjected to metagenomic analyses [3]. Results revealed the presence of ~ 11,000 DNA viruses and indicated that the microbiome of built-environment surfaces is distinctly different from that of other environments, such as the human body and soil. The METACoV project, derived from this project, aimed to characterize the changes in the urban microbiome and RNA virome, which includes the SARS-CoV-2 virus, during the COVID-19 pandemic via shotgun metatranscriptomics (total RNA-seq) [4].

However, RNA virome analysis of built-environment surfaces is associated with several challenges. First, the amount of RNA obtained is markedly lower (in the range of picograms or less) than the input recommendations of standard library preparation kits (in the range of nanograms) owing to the extremely low biomass detected [5–7]. Standard protocols for library construction usually recommend starting with a nanogram order of total RNA. However, several researchers have successfully prepared high-quality libraries from lower amounts of input RNA (250–500 pg) than those recommended by the manufacturers [8, 9]. Second, since most shotgun DNA sequences on built-environment surfaces are of bacterial origin, followed by eukaryotes such as fungi and humans [5, 6], abundant rRNA might reduce the sensitivity of RNA virome analysis. Several studies that detected SARS-CoV-2 in human clinical specimens via metatranscriptomics reported that human or bacterial rRNA depletion can improve viral genome detection [10–14]. Owing to the issues and sequencing costs of RNA analysis, no reports of metatranscriptomic RNA virome analysis from built-environment surfaces are currently available, to our knowledge.

Therefore, in this pilot study, we evaluated the impact of rRNA depletion methods on the quality of sequencing libraries, community composition, and sensitivity of virus detection using mock community RNA (0.1–10 ng) and a melamine-coated table surface RNA (< 1 ng), which had RNA amounts below the recommended input RNA amount (< 5 ng) for the library preparation kit used here. We also investigated the detection limit of SARS-CoV-2 using serial dilutions of synthetic viral RNA spiked into RNA samples from the table surface.

## Materials and methods

**Fig. 1:**
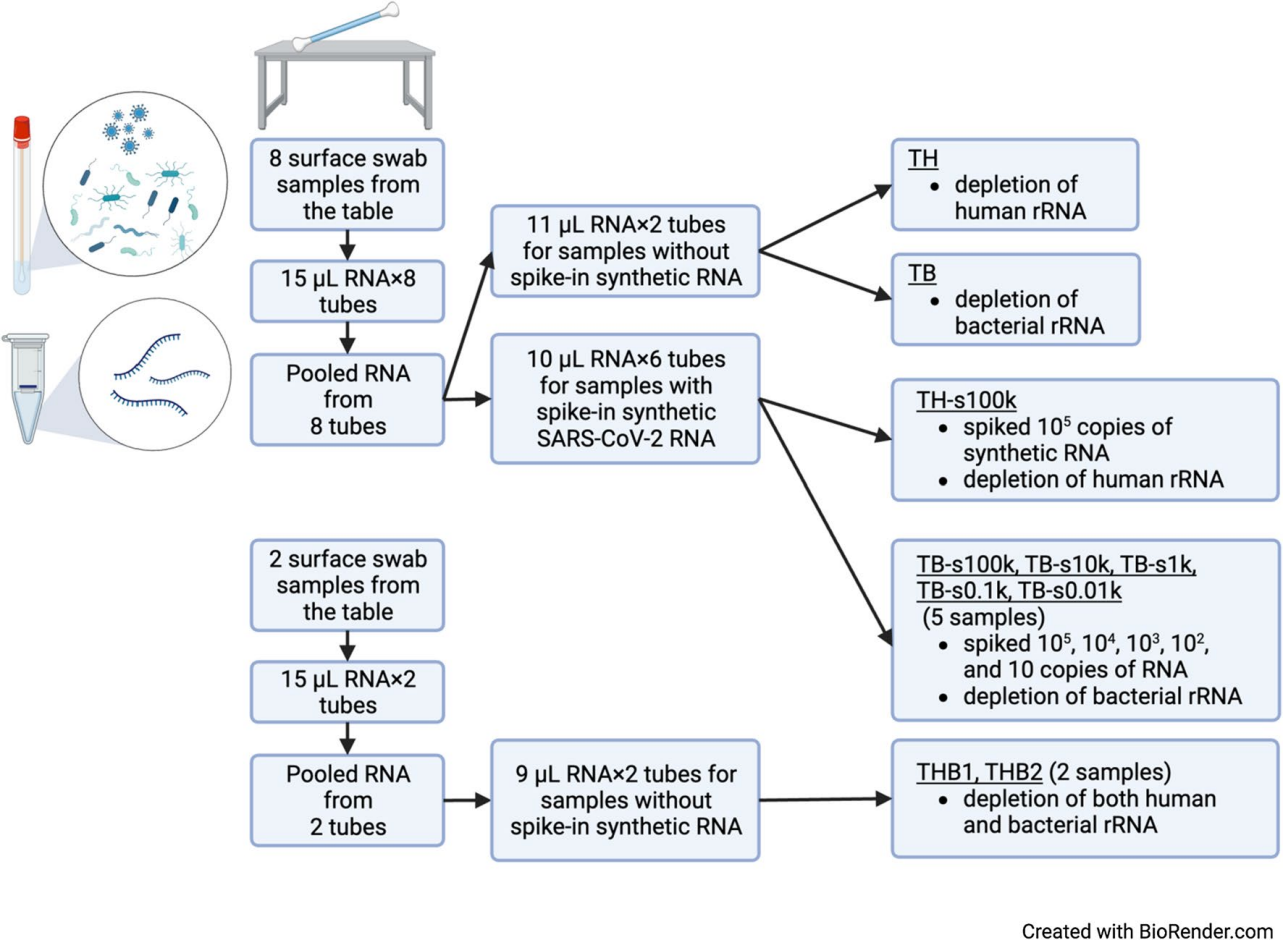
The sampling, RNA extraction, and pooling strategy. Underlines correspond to sample names in the main text. Figure created with BioRender.com

Please refer to Additional file 1 for sample collection, RNA extraction, rRNA depletion, library preparation and sequencing, and bioinformatics methods. The sampling, RNA extraction, and pooling strategy is illustrated in Fig. 1.

## Results and discussion

### Effects of mock community RNA input amount on library preparation

We used a combination of NEBNext rRNA Depletion Kit for rRNA depletion and NEBNext Ultra II Directional RNA Library Prep Kit for metatranscriptomic library preparation. The minimum recommended combined amount of total RNA for these kits is 5 ng. To test the feasibility of library preparation from RNA amounts below the protocol requirements, total RNA extracted from ZymoBIOMICS Microbial Community Standard cells was serially diluted to 10, 1.0, and 0.1 ng, and rRNA depletion and library preparation were performed. Since this mock community primarily consisted of bacteria, rRNA depletion kits for human RNA were used to preserve the abundant bacterial rRNA.

All three samples (10, 1, and 0.1 ng) were successfully prepared for library construction and sequenced. Adapter concentration and number of PCR cycles were optimally adjusted, resulting in high-quality reads (Additional file 2: Table S1). Read duplication rates increased with decreasing amounts of input RNA. Higher duplicate rates indicate lower RNA complexity in the samples, which can reflect the presence of abundant bacterial rRNA that was not depleted (Additional file 2: Table S1) and the insufficient amount of input RNA, especially in the 0.1 ng sample. We subsequently assessed the bias of reduced RNA input on the species relative abundance estimate, and no clear differences in taxonomic compositions were observed among the three samples (Additional file 2: Table S2). Since information on the RNA composition ratios of each species in the mock community was not provided by the manufacturer, comparisons with theoretical content were not possible. However, when results for 10 ng RNA samples were compared with those for the other samples, no marked differences were observed, except for a slight difference in the percentage of *Bacillus subtilis*. This indicates that the combined use of the rRNA depletion kit and library preparation kit can produce high-quality libraries, even when the RNA input is reduced to 0.1 ng (100 pg). Our results are consistent with the 250–500 pg of RNA input reported previously [8, 9].

### Library preparation from table surface samples, rRNA depletion efficiency, and community composition

To evaluate the feasibility of library preparation from built-environment surface RNA samples with less than the recommended RNA amount for the library preparation kit, we used RNA samples from a melamine-coated table used daily by students. Simultaneously, we evaluated the impact of human or bacterial rRNA depletion on community composition and virus detection sensitivity. We spiked serial dilutions of synthetic SARS-CoV-2 RNA (10^1^–10^5^ copies) into RNA samples from the table, followed by rRNA depletion of either human or bacterial rRNA, and then performed library preparation. Abundant bacteria were assumed to be present on the table surface [5, 6]; therefore, bacterial rRNA depletion was performed for five samples representing the full range of spike-in levels evaluated. An additional human rRNA-depleted sample spiked with 10^5^ copies of SARS-CoV-2 was also prepared.

Although the RNA concentrations of the table surface samples were below the limit of detection of Qubit RNA HS Assay Kit (0.25 ng/µL), all samples (except the negative control) allowed generation of sufficient libraries for sequencing (Table 1). Thus, the library concentration can be used as a proxy for the RNA concentration initially extracted from each sample, and since the library concentration obtained from the table sample is approximately three times higher than that obtained from 0.1 ng of mock RNA (Additional file 2: Table S1 and Table 1), the amount of RNA in the table sample was estimated to be ~ 0.3 ng.

**Table 1:** Library preparation and sequencing summary metrics

Sample	Spiked synthetic RNA copies per library	rRNA depletion	Average library size (bp)	Library molarity (nM)	No. of paired reads	Passed Filter (%)	Duplication rate (%)	GC content	% Eukaryotic rRNA reads (18S, 28S)	% Bacterial rRNA reads (16S, 23S)	% Non-ribosomal RNA reads
TH	NA	Human	335	45.4	9,431,639	96.4	55.4	52.8	4.3	85.8	9.9
TH-s100k	10^5^	Human	331	43.1	11,565,081	97.7	59.8	52.7	4.5	86.9	8.6
TB	NA	Bacteria	329	48.0	10,890,472	96.5	57.9	52.3	89.2	1.1	9.6
TB-s100k	10^5^	Bacteria	329	61.3	9,456,268	97.0	57.2	52.2	88.4	0.8	10.8
TB-s10k	10^4^	Bacteria	330	43.1	10,113,548	97.0	59.3	52.3	88.0	1.2	10.8
TB-s1k	10^3^	Bacteria	331	63.7	10,001,537	97.5	56.6	52.2	87.8	0.8	11.3
TB-s0.1 k	10^2^	Bacteria	337	61.3	9,888,461	97.3	55.1	52.3	88.9	0.9	10.2
TB-s0.01 k	10^1^	Bacteria	325	43.8	10,581,258	97.5	61.9	52.4	89.0	0.8	10.2
NH	NA	Human	276	2.2	5,429,140	53.0	63.4	50.9	11.6	28.5	59.0
NB	NA	Bacteria	309	2.8	6,926,797	85.4	75.1	52.4	23.9	29.2	46.4

*TH* table surface sample depleted of human rRNA, *TB* table surface sample depleted of bacterial rRNA, *s100k* spiked 10^5^ copies of synthetic RNA, *NH* negative control depleted of human rRNA, *NB* negative control depleted of bacterial rRNA, *NA* not applicable

All samples, including negative controls, were successfully sequenced (Table 1). The average percentage of high-quality reads that passed quality filtering was 97.1%, with read duplication rates of 55.4–61.9%. The rRNA read percentages showed clear differences between the two depletion methods. The percentage of eukaryotic rRNA reads in both human and bacterial rRNA-depleted samples averaged 4.4% and 88.5%, respectively. Conversely, the percentage of bacterial rRNA reads in human and bacterial rRNA-depleted samples averaged 86.3% and 0.9%, respectively.

We evaluated the effect of different rRNA depletion methods on community composition (Table 2). The estimated relative abundance of eukaryotes in human and bacterial rRNA-depleted samples averaged 19.9% and 89.1%, respectively, while that of bacteria in human and bacterial rRNA-depleted samples averaged 79.8% and 10.6%, respectively, a percentage of organisms that corresponds to that for the rRNA depletion method. When compared with that of the non-spiked samples (TH vs. TB samples), the estimated viral occupancy of bacterial rRNA-depleted samples was slightly higher than that of human rRNA-depleted samples (Table 2, TH: 0.061% vs. TB: 0.076%). Even after rRNA depletion using either method, the percentage of non-rRNA reads was ~ 10% (Table 1).

**Table 2:** Sequence read classification and mapping to the SARS-CoV-2 genome

Sample	% Classified reads by Kraken2	Distribution of reads classified			Identification of SARS-CoV-2				
		% Eukaryota	% Bacteria	% Viruses	No. of classified reads by Bracken	No. of mapped reads by BWA-MEM	Average depth of genome coverage	% Genome covered	% Reads mapped to 3'UTR
TH	95.1	19.9	79.8	0.061	0	81	0.1	0.1	100.0
TH-s100k	95.7	19.8	79.7	0.319	12,693	51,083	240.2	100.0	1.6
TB	95.0	88.8	11.0	0.076	0	84	0.1	0.1	100.0
TB-s100k	94.6	89.1	9.5	1.412	65,368	55,076	260.6	100.0	1.1
TB-s10k	94.4	88.8	11.0	0.201	5685	6236	28.2	98.0	3.7
TB-s1k	94.6	89.3	10.6	0.087	421	941	2.7	43.3	37.5
TB-s0.1 k	94.6	89.3	10.6	0.077	0	86	0.2	3.3	69.8
TB-s0.01 k	94.6	89.3	10.6	0.081	0	72	0.1	0.1	100.0
NH	87.3	29.6	70.4	0.054	0	1376	1.2	0.9	99.9
NB	86.6	37.9	61.9	0.036	0	1437	1.2	0.2	94.0

**Fig. 2:**
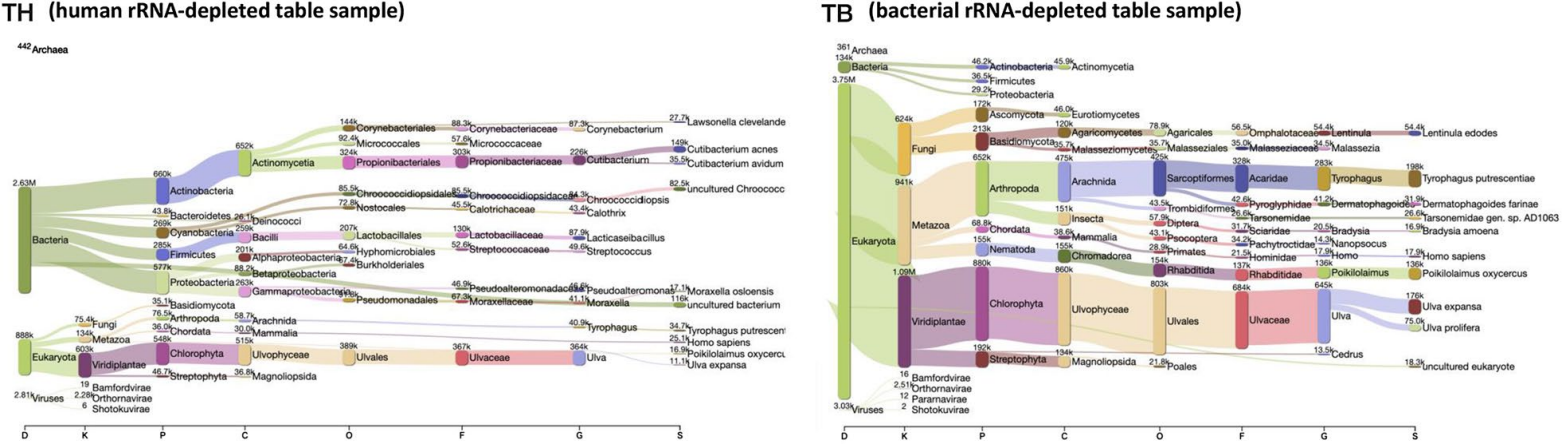
Sankey diagrams of the community composition of the table surface. The number of reads is proportional to the flow width. The corresponding number of each node represents the k-mer hit number. Domain (D), kingdom (K), phylum (P), class (C), order (O), family (F), genus (G), or species (S) was used as the rank code. TH, table surface sample depleted of human rRNA; TB, table surface sample depleted of bacterial rRNA

We then focused on the community composition of the table surface (Fig. 2 and Additional file 2: Table S3). In human rRNA-depleted samples, the commensal skin bacterium *Cutibacterium acnes* was the most abundant. This is likely because table surfaces frequently come in contact with the human skin. In contrast, in bacterial rRNA-depleted samples, mites and nematodes (*Tyrophagus putrescentiae*, *Poikilolaimus oxycercus*) and seaweed (*Ulva expansa/prolifera*) were the most abundant. Since seaweed is commonly used in Japanese foods, it may have originated from meal contaminants present on table surfaces.

This demonstrates that metatranscriptome analysis may be performed on RNA from table surfaces using a standard library preparation kit. Additionally, differences in target species for the rRNA depletion method affected community composition and viral detection sensitivity.

### Effects of rRNA depletion on sensitivity of SARS-CoV-2 RNA spiked into RNA samples

To assess the detection limit of SARS-CoV-2 RNA, we evaluated the number of SARS-CoV-2 reads obtained for the depleted samples of spiked human and bacterial rRNA using a k-mer (Kraken2–Bracken) and an alignment-based (BWA-MEM) approach. Kraken2–Bracken results indicated that no reads were detected in the negative control and non-spiked samples; however, SARS-CoV-2 reads were detected at > 10^3^ copies in bacterial rRNA-depleted samples, with the number of reads increasing almost 10 fold with an increase in copy number (Table 2). When compared with samples with 10^5^ copies of SARS-CoV-2 spiked-in (TH-s100k vs. TB-s100k), the bacterial rRNA-depleted sample (TB-s100k) showed a ~ 5 fold increase in the number of SARS-CoV-2 reads compared with the human rRNA-depleted sample (TH-s100k, Bracken reads: 65,368 vs. 12,693).

While Kraken2-Bracken did not detect any reads classified as SARS-CoV-2 in negative controls and non-spiked samples, BWA-MEM results revealed 81–1,437 reads mapped to the SARS-CoV-2 reference genome in those samples, signifying false positive results. Majority of these reads mapped to a polyA-containing region in the 3′ UTR of low complexity (Additional file 2: Table S4), suggesting that they were false positives. Similarly, 72 (TB-s0.01 k) and 86 (TB-s0.1 k) reads detected with samples spiked-in with less than 10^3^ copies were attributed to false positive results. Compared with those in the samples with 10^5^ copies of SARS-CoV-2 (TH-s100k vs. TB-s100k), SARS-CoV-2 reads were slightly higher in the bacterial rRNA-depleted sample, although the difference was smaller than that obtained using Kraken2-Bracken. When comparing bacterial rRNA-depleted samples, SARS-CoV-2 genome coverage was 43.3% and 98.0% for the 10^3^ (TB-s1k) and 10^4^ (TB-s10k) copies of SARS-CoV-2 spiked-in samples, respectively (Table 2 and Additional file 3: Figure S1). The average depth of genome coverage was 2.7 × and 28.2 × for the 10^3^ (TB-s1k) and 10^4^ (TB-s10k) copies of SARS-CoV-2 spiked-in samples, respectively.

In summary, at least 10^4^ copies are required to obtain the average genome coverage depth (~ 30 ×) and genome coverage (> 98%) needed to detect mutations in the SARS-CoV-2 genome in this experimental system, and the detection limit for SARS-CoV-2 RNA is 10^3^ copies. This value corresponds to that reported in previous studies on SARS-CoV-2 surface swabbing using qRT-PCR [15]. Although our study used synthetic RNA and theirs used inactivated viral particles, they reported that a minimum of 1,000 viable viral particles per 25 cm^2^ surface is required to ensure successful virus recovery and detection.

### Efficiency of simultaneous depletion of both human and bacterial rRNA

Depletion of both human- and bacteria-derived rRNA may further increase the number of virus-derived reads; therefore, we employed simultaneous depletion of both human and bacterial rRNA (see Materials and Methods). The percentage of non-rRNA reads was 61.6% (THB1) and 62.6% (THB2) in the simultaneously depleted samples (Additional file 2: Table S5), markedly higher than that in samples depleted of either human (9.9%) or bacterial (9.6%) rRNA (Table 1). The percentage of viral occupancy was 0.259% (THB1) and 0.290% (THB2) in the simultaneously depleted samples (Additional file 2: Table S5), a 3.4-fold (THB1) and 3.8-fold (THB2) increase compared to that in the bacterial rRNA-depleted samples (0.076%; Table 2).

This demonstrates that simultaneous depletion of both human- and bacteria-derived rRNAs further increased the number of virus-derived reads. However, human RNA was not the only major source of eukaryotic RNA on the table surface (Additional file 3: Figure S2), suggesting that depletion of rRNAs of non-human eukaryotic origin is important for further improving virus detection sensitivity.

## Conclusions

Here, we demonstrated that metatranscriptome analysis can be performed on RNA from table surfaces, representative of built-environment surfaces, using a standard library preparation kit by changing the adapter concentration and number of PCR cycles. The rRNA depletion method performed also influenced community composition and virus detection sensitivity.

## Limitations

The built-environment surface targeted here was limited to one table, and the effects of rRNA depletion on detecting a small number of virus-derived reads might be considerably different with other surfaces. Owing to the limited amount of table surface-derived RNA that could be prepared, verifying reproducibility under each condition was difficult. Additionally, because the viral synthetic RNA used was spiked into the sample RNA after extraction, the detection sensitivity of the viral synthetic RNA may have been biased toward high sensitivity, compared to more realistic methods that absorb synthetic RNA into the swab during sampling.

## Supplementary Information

**Additional file 1:** Materials and methods

**Additional file 2: Table S1** Library preparation and sequencing summary metrics of the mock community samples. **Table S2** Species relative abundance of the mock community samples estimated using Bracken. **Table S3** Species relative abundance of the table and negative control samples estimated using Bracken. **Table S4 **The number of reads and coverage for each SARS-CoV-2 genomic feature (e.g., genes, 3′ UTR). **Table S5** Library preparation and sequencing summary metrics of both human and bacterial rRNA-depleted samples.

**Additional file 3: Figure S1 **Graphical view of reads mapped to the reference genome of SARS-CoV-2. The blue tracks represent the SARS-CoV-2 reference strain, and the subsequent four tracks represent Bam files of bacterial rRNA-depleted samples spiked with synthetic RNA of SARS-CoV-2 (s100k, 10^5^; s10k, 10^4^; s1k, 10^3^; s0.1k, 10^2^; copies of SARS-CoV-2 RNA). **Figure S2 **Sankey diagrams of the Kraken 2 report based on human and bacterial rRNA-depleted samples (THB1 and THB2).

## Data Availability

The sequenced raw reads used for this study were deposited in the DNA Data Bank of Japan Sequence Read Archive (DRA), the National Center for Biotechnology Information Sequence Read Archive (SRA), and the European Bioinformatics Institute Sequence Read Archive (ERA) under the accession numbers DRA014951 and DRA015134.

## Abbreviations

DRS: DNA/RNA shield
NTC: No template control
