# Supplementary material for: Evaluation of rRNA depletion methods for capturing the RNA virome from environmental surfaces
Source: BMC Res Notes. 2023 Jul 7;16:142. doi: 10.1186/s13104-023-06417-9 (PMC10326927; doi:10.1186/s13104-023-06417-9)
Supplement: Supplementary file 1 — Additional file 1: Materials and methods [file 13104_2023_6417_MOESM1_ESM.docx]

**Additional file 1: Materials and methods**

Descriptions of the sample collection, RNA extraction, ribosomal RNA depletion, library preparation and sequencing, and bioinformatics methods.

**Sample collection**

A melamine resin coated table, frequently used by students at a university campus in Tokyo, was swabbed and a total of 10 samples were collected in the two experiments described below. After RNA was extracted from the individual swabs collected, the RNA solutions were pooled and used for each experiment. The sampling, RNA extraction and pooling strategy was illustrated in Figure 1. Prefilled 1 mL barcoded tubes containing 400 µL of DNA/RNA Shield (DRS; Zymo Research Corp., Germany) preservative provided by Mason Lab at Weill Cornell Medicine as a part of MetaSUB projects, were used for each sample. Isohelix Mini Swab (MS-03, Cell Projects Ltd, UK) was immersed in DRS for 15 s prior to sampling to ensure the swab was sufficiently moistened. The moistened swab was dragged firmly across the surface for a total of 3 min to ensure the highest yield using both sides and different angles.

For human rRNA-depleted samples and bacterial rRNA-depleted samples, we collected 12 surface swab samples from the table. For an additional experiment of simultaneous depletion of both human and bacterial rRNA, we collected two surface swab samples from the same table at different times from the aforementioned sampling.

Once the surface was sampled, the swabs were immediately placed in the pre-filled tubes. In addition to the sample swabs, a single swab was immersed in a commercial whole-cell mock community consisting of eight bacteria and two yeasts cells (ZymoBIOMICS Microbial Community Standard, D6300). These samples were stored at -80 °C until nucleic acid extraction.

**RNA extraction**

Total nucleic acid extraction was performed according to Promega's nucleic acid purification application notes (Viral RNA Purification and Detection from Environmental Surfaces, <https://promega.widencollective.com/portals/7awf3axg/AppNotesNAP>). Samples on the swab tip were vortexed in DRS for 2 min, and 200 μL of the DRS solution was subjected to nucleic acid extraction using Maxwell PureFood GMO and Authentication Kit on a Maxwell RSC Instrument (Promega Corp., Madison, WI). Total nucleic acid was eluted in 100 μL nuclease-free water and stored in a -80°C freezer until RNA extraction. RNA was further purified from the 100 μL of total nucleic acid solution using a RNA clean & concentrator-5 kit with DNase I treatment (Zymo Research Corp., Germany) and eluted in 15 μL nuclease-free water. For human rRNA-depleted samples and bacterial rRNA-depleted samples, the elutions of eight RNA table surface samples were pooled. For both human and bacterial rRNA-depleted samples, the elutions of two RNA table surface samples were pooled. The RNA concentration was determined using a Qubit 3 Fluorometer with the Qubit RNA HS Assay Kit (Life Technologies, Thermo Fisher Scientific Inc.) and immediately stored at -80°C.

**Preparation of human and bacterial rRNA-depleted samples**

For rRNA depletion, either the NEBNext rRNA Depletion Kit v2 for Human/Mouse/Rat (E7405) or for Bacteria (E7860) was used according to the manufacturer's instructions. 11 µL total RNA was used as the input amount. Total RNA for rRNA depletion was prepared as follows: the mock community RNA concentration was determined as 136 ng/µL and was serially diluted to 10, 1.0, and 0.1 ng in 11 µL nuclease-free water. The RNA concentrations of the pooled table surface-derived samples were below the detection limit of the Qubit RNA HS Assay Kit (0.25 ng/µL), hence they were used directly for rRNA depletion without concentration adjustment. For samples spiked with synthetic SARS-CoV-2 RNA, Twist Synthetic SARS-CoV-2 RNA Control 1 (Twist Bioscience, South San Francisco, CA, USA, #MT007544.1) was serially diluted to 10^5^, 10^4^, 10^3^, 10^2^, and 10 copies/μL with nuclease-free water. The pooled table RNA was divided into six aliquots of 10 μL each, and each sample was added to 1 μL of diluted synthetic SARS-CoV-2 RNA. For samples without spike-in synthetic RNA, 11 μL pooled RNA and 11 μL sterile water was prepared as a negative control (NTC, no template control). For the mock community (mainly composed of bacteria), rRNA depletion was performed using the NEBNext rRNA Depletion Kit v2 for Human/Mouse/Rat specifically to retain the bacterial rRNA. For table surface samples, Human/Mouse/Rat kit or the same kit for bacteria were used for each condition to compare the effects of rRNA depletion methods (Table 1).

**Preparation of both human and bacterial rRNA depleted samples**

The pooled table RNA from two swabs was divided into two aliquots of 9 μL each (synthetic SARS-CoV-2 RNA was not spiked). Depletion of rRNA and library preparation were performed in duplicate. Simultaneous depletion of both human and bacterial rRNA was performed using a combination of two NEBNext rRNA Depletion Kits (Human/Mouse/Rat and Bacteria) according to the manufacturer’s instructions with the following modifications: 9 μL of each sample was subject to probe hybridization to RNA using 2 μL NEBNext v2 rRNA Depletion Solution provided in the Human/Mouse/Rat kit, 2 μL NEBNext Bacterial rRNA Depletion Solution provided in the bacteria kit, 2 μL NEBNext Bacterial rRNA Depletion Solution, and 2 μL NEBNext Probe Hybridization Buffer.

**Library preparation and sequencing**

Libraries were then prepared from rRNA depleted samples (i.e., those containing mRNA, tRNA, etc.) using the NEBNext Ultra II Directional RNA Library Prep Kit (E7760) and NEBNext Multiplex Oligos for Illumina (E7335 and E7500) according to the manufacturer’s instructions with the following modifications: RNA was fragmented at 94 °C for 7 min prior to first-strand synthesis. During the adaptor ligation step, NEBNext Adaptor was diluted 100-fold for the 10 ng and 1 ng mock community samples and 500-fold for the 0.1 ng mock community and table surface samples. The number of PCR cycles for amplification of adapter-ligated DNA was 16 for the 10 ng and 1 ng mock communities, and 20 for the other samples. Individual libraries were quantified using an Agilent Tapestation 4200 with High Sensitivity D1000 ScreenTape (Agilent Technologies, Santa Clara, CA, USA). Individual libraries were then pooled and quantified using KAPA Library Quantification Kit Illumina Platforms (Kapa Biosystems, Wilmington, MA, USA). For human rRNA-depleted samples and bacteria rRNA-depleted samples, the pooled library was sequenced using 151 bp paired-end on the NextSeq 500 using the 300 cycles NextSeq 500/550 Mid Output Kit v2.5 (Illumina, San Diego CA). For both human and bacterial rRNA depleted samples, the pooled library was sequenced using 151 bp paired-end on the MiniSeq using the 300 cycles MiniSeq Mid Output Kit (Illumina, San Diego CA).

**Bioinformatics**

The FASTQ files were then uploaded to the local Galaxy server [1] for further processing. All samples including NTC yielded > 5 million (M) read pairs (Supplementary Table 1 and Table 1), and reads were randomly subsampled to 10 M reads (5 M read pairs) using seqtk_sample (Galaxy Version 1.3.2, https://github.com/lh3/seqtk) with default settings. Subsampled reads were preprocessed using fastp (Galaxy Version 0.23.2+galaxy0) [2] to filter low-quality reads and remove adapters with default settings. MultiQC (Galaxy Version 1.11+galaxy0) [3] was then used for result aggregation from fastp pre-processed reads into a single report.

The proportion of ribosomal and non-ribosomal RNA reads to total pre-processed reads was calculated using SortMeRNA (Galaxy Version 2.1b.6) [4] and several rRNA databases (silva-arc-16s-id95, silva-arc-23s-id98, silva-bac-16s-id90, silva-bac-23s-id98, silva-euk-18s-id95, silva-euk-28s-id98, rfam-5s-database-id98, and rfam-5.8s-database-id98) with the “generate statistics” parameter changed to “Yes”.

All pre-processed reads, following the preprocess, were assigned taxonomy using Kraken2 (Galaxy Version 2.1.1+galaxy1) [5] against the NCBI non-redundant nucleotide database (built in May 2021) with the “confidence” parameter changed to “0.05”. The domain- and species-level taxonomic abundancy values were estimated using Bracken (Galaxy Version 2.7.1+galaxy0) [6] based on the read counts from Kraken2. The Kraken-classified report was visualized using Pavian (https://github.com/fbreitwieser/pavian) [7].

All pre-processed reads were also mapped and aligned against the complete genome sequence of Severe Acute Respiratory Syndrome Coronavirus 2 isolate Wuhan-Hu-1 (RefSeq ACCESSION: NC_045512.2) using BWA-MEM (Galaxy Version 0.7.17.1) [8] with default settings and filtered for high-quality mapping (MapQuality ≥  30) using BAM tools (Galaxy Version 2.4.1) [9]. The average depth of the reference genome was calculated using QualiMap BamQC tool (Galaxy Version 2.2.2d+galaxy1) [10]. Genome coverage was calculated as the percentage of genomic positions covered by at least one read. The number of reads and coverage for each genomic feature (e.g., genes, 3' UTR, etc.) were also calculated based on each alignment using the annotateBed tool in BEDTools (Galaxy Version 2.29.0) [11]. The Integrative Genomics Viewer (IGV) software (http://software.broadinstitute.org/software/igv/home) was used to visualize alignments in bam format [12].

**References**

1. Afgan E, Baker D, Batut B, van den Beek M, Bouvier D, Čech M, et al. The Galaxy platform for accessible, reproducible and collaborative biomedical analyses: 2018 update. Nucleic Acids Res. 2018;46 Web Server issue:gky379-.

2. Chen S, Zhou Y, Chen Y, Gu J. fastp: an ultra-fast all-in-one FASTQ preprocessor. Bioinformatics. 2018;34:i884–90.

3. Ewels P, Magnusson M, Lundin S, Käller M. MultiQC: summarize analysis results for multiple tools and samples in a single report. Bioinformatics. 2016;32:3047–8.

4. Kopylova E, Noé L, Touzet H. SortMeRNA: fast and accurate filtering of ribosomal RNAs in metatranscriptomic data. Bioinformatics. 2012;28:3211–7.

5. Wood DE, Lu J, Langmead B. Improved metagenomic analysis with Kraken 2. Genome Biol. 2019;20:257.

6. Lu J, Breitwieser FP, Thielen P, Salzberg SL. Bracken: estimating species abundance in metagenomics data. Peerj Comput Sci. 2017;3:e104.

7. Breitwieser FP, Salzberg SL. Pavian: interactive analysis of metagenomics data for microbiome studies and pathogen identification. Bioinformatics. 2019;36:1303–4.

8. Li H. Aligning sequence reads, clone sequences and assembly contigs with BWA-MEM. Arxiv. 2013.

9. Barnett DW, Garrison EK, Quinlan AR, Strömberg MP, Marth GT. BamTools: a C++ API and toolkit for analyzing and managing BAM files. Bioinformatics. 2011;27:1691–2.

10. Okonechnikov K, Conesa A, García-Alcalde F. Qualimap 2: advanced multi-sample quality control for high-throughput sequencing data. Bioinformatics. 2016;32:292–4.

11. Quinlan AR, Hall IM. BEDTools: a flexible suite of utilities for comparing genomic features. Bioinformatics. 2010;26:841–2.

 12. Robinson JT, Thorvaldsdóttir H, Winckler W, Guttman M, Lander ES, Getz G, et al. Integrative genomics viewer. Nat Biotechnol. 2011;29:24–6.
