# Supplementary material for: Evaluation of rRNA depletion methods for capturing the RNA virome from environmental surfaces
Source: BMC Res Notes. 2023 Jul 7;16:142. doi: 10.1186/s13104-023-06417-9 (PMC10326927; doi:10.1186/s13104-023-06417-9)
Supplement: Supplementary file 3 — Additional file 3: Figure S1 Graphical view of reads mapped to the reference genome of SARS-CoV-2. The blue tracks represent the SARS-CoV-2 reference strain, and the subsequent four tracks represent Bam files of bacterial rRNA-depleted samples spiked with synthetic RNA of SARS-CoV-2 (s100k, 105; s10k, 104; s1k, 103; s0.1k, 102; copies of SARS-CoV-2 RNA). Figure S2 Sankey diagrams of the Kraken 2 report based on human and bacterial rRNA-depleted samples (THB1 and THB2). [file 13104_2023_6417_MOESM3_ESM.pdf]

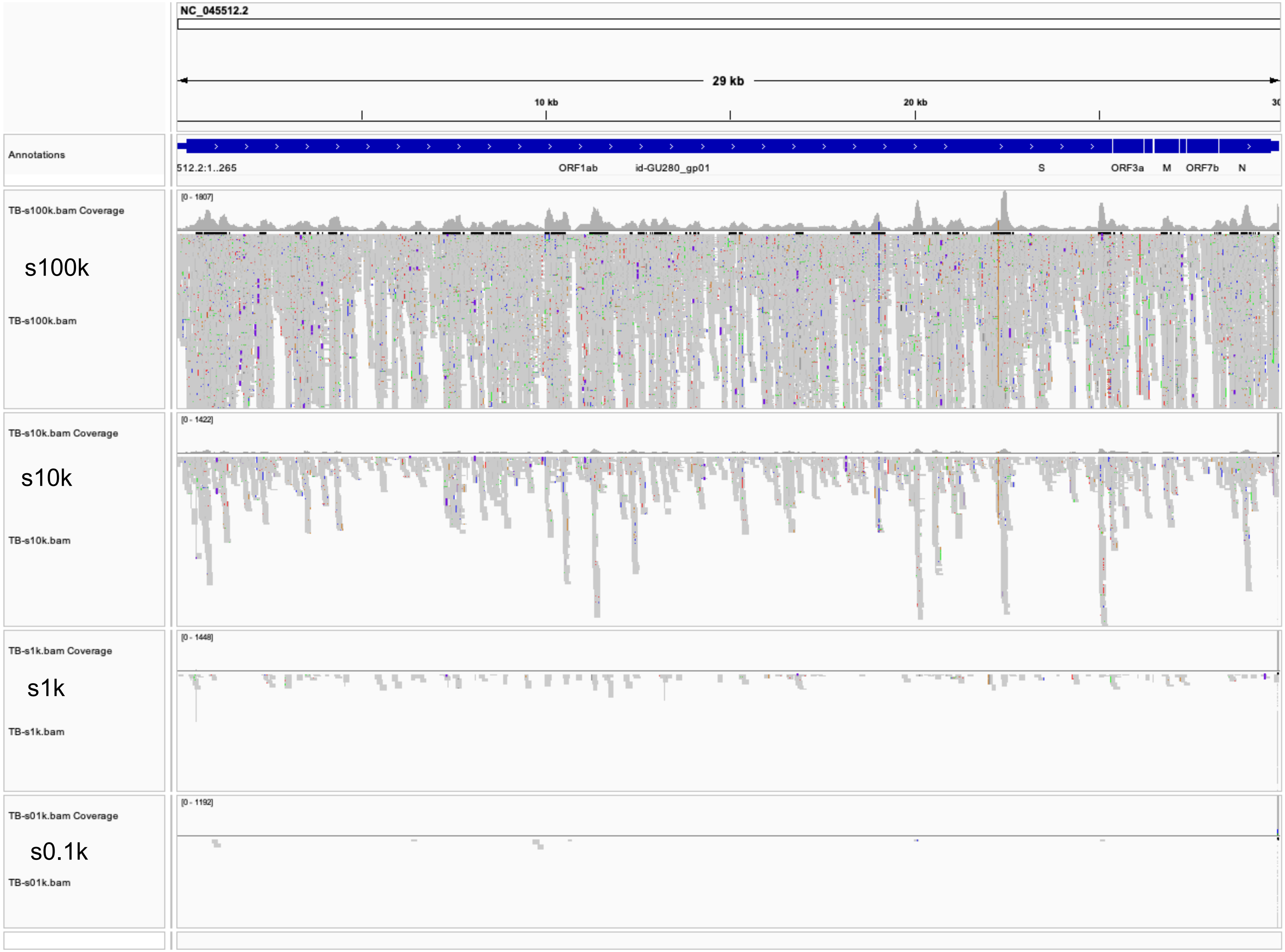

Figure S1. Graphical view of reads mapped to the reference genome of SARS-CoV-2. The blue tracks represent the SARS-CoV-2 reference strain, and the subsequent four tracks represent Bam files of bacterial rRNA-depleted samples spiked with synthetic RNA of SARS-CoV-2 (s100k, 10^5; s10k, 10^4; s1k, 10^3; s0.1k, 10^2; copies of SARS-CoV-2 RNA).

THB1

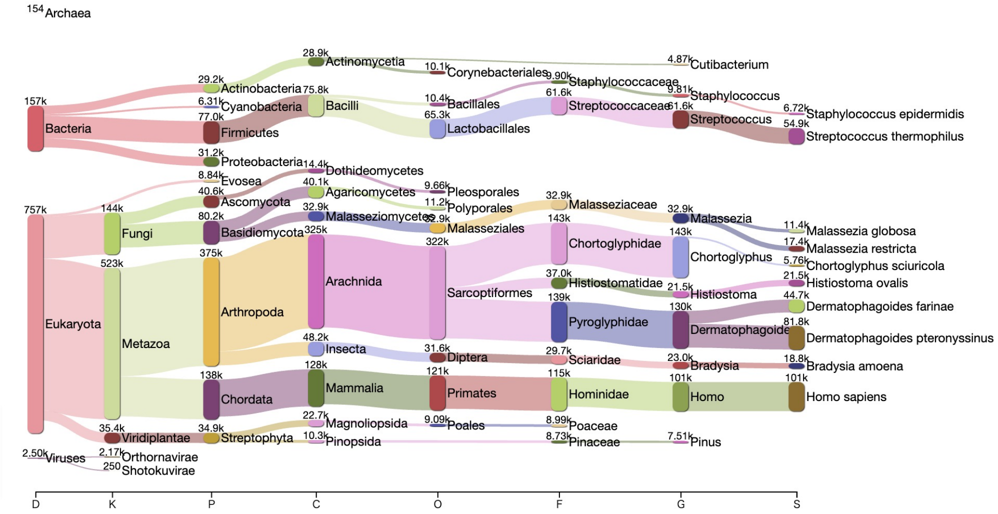

THB2

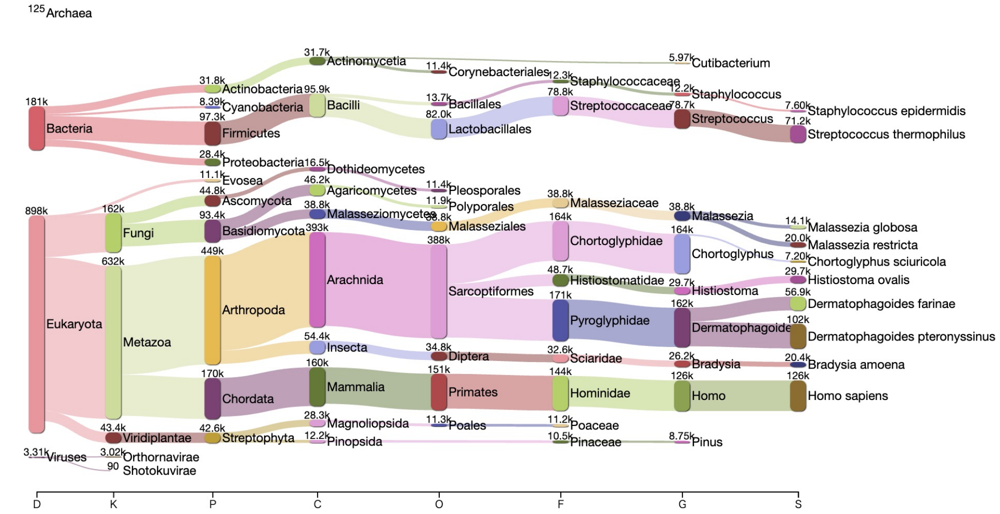

Figure S2. Sankey diagrams of the Kraken 2 report based on human and bacterial rRNA-depleted samples (THB1 and THB2).
